# Temporal patterns of charcoal burning suicides among the working age population in Hong Kong SAR: the influence of economic activity status and sex

**DOI:** 10.1186/1471-2458-12-505

**Published:** 2012-07-06

**Authors:** Chi-kin Law, Candi MC Leung

**Affiliations:** 1 Hong Kong Institute of Asia-Pacific Studies, The Chinese University of Hong Kong, Shatin, Hong Kong; 2 Australian Institute for Suicide Research and Prevention, Griffith University, Griffith, Australia; 3 Department of Psychology, The University of Hong Kong, Pokfulam, Hong Kong

**Keywords:** Temporal variation, charcoal burning, suicide prevention, time pattern, seasonal risk

## Abstract

**Background:**

Charcoal burning in a sealed room has recently emerged as the second most common suicide means in Hong Kong, causing approximately 200 deaths each year. As charcoal burning suicide victims have a unique sociodemographic profile (i.e., predominantly economically active men), they may commit suicide at specific times. However, little is known about the temporal patterns of charcoal burning suicides.

**Methods:**

Suicide data from 2001 to 2008 on victims of usual working age (20–59) were obtained from the registered death files of the Census and Statistics Department of Hong Kong. A total of 1649 cases of charcoal burning suicide were analyzed using a two-step procedure, which first examined the temporal asymmetries in the incidence of suicide, and second investigated whether these asymmetries were influenced by sex and/or economic activity status. Poisson regression analyses were employed to model the monthly and daily patterns of suicide by economic activity status and sex.

**Results:**

Our findings revealed pronounced monthly and daily temporal variations in the pattern of charcoal burning suicides in Hong Kong. Consistent with previous findings on overall suicide deaths, there was an overall spring peak in April, and Monday was the common high risk day for all groups. Although sex determined the pattern of variation in charcoal burning suicides, the magnitude of the variation was influenced by the economic activity status of the victims.

**Conclusion:**

The traditional classification of suicide methods as either violent or nonviolent tends to elide the temporal variations of specific methods. The interaction between sex and economic activity status observed in the present study indicates that sex should be taken into consideration when investigating the influence of economic activity status on temporal variations of suicide. This finding also suggests that suicide prevention efforts should be both time- and subgroup-specific.

## Background

Temporal variation in suicide deaths is an important topic in suicide research worldwide. Identifying the most probable timing of suicidiality will help to improve intervention and prevention efforts. Charcoal burning in a sealed room has recently emerged as the second most common suicide method in Hong Kong [1-5]. Being a relatively painless and inexpensive method, charcoal burning has rapidly increased the suicide rates in the past few years [6]. However, no prior research has examined the temporal pattern of suicides by charcoal burning.

Previous studies consistently showed asymmetrical distributions of suicide deaths. For both sexes, major peaks were found in late spring or summer (“Spring fever”) [7-12], whereas a nadir was noted in December (winter) [13]. Compared with monthly patterns, the daily (referred to as “weekday” or “weekly” in the literature) pattern of suicides is relatively under-researched, particularly in the Hong Kong context. Notwithstanding the limited number of studies, the findings on the daily pattern of suicides were similar to those observed in the related studies, with marked peaks on Mondays and nadirs on weekends [14,15]. Thus, charcoal burning suicides may also have temporal variations if they follow a similar pattern.

However, being a non-violent suicide method, charcoal burning is often assumed to have no obvious temporal patterns. In some seasonality studies, suicides by violent methods (e.g. jumping and hanging) showed more marked temporal variations when compared with nonviolent suicides (e.g. poisoning) [8,16,17]. This may suggest that charcoal burning suicides do not have obvious temporal variations in either monthly or daily distributions. Accordingly, the recent surge in suicides by charcoal burning would lead to a smoothing of the temporal variations in suicide in Hong Kong.

The unique sociodemographic profile of the victims of charcoal burning suicide suggests that they may tend to commit suicide at specific times. Charcoal burning suicide victims were more likely to be economically active and over-indebted than other suicide victims [1]. Although economic activity status had no influence on the monthly pattern of overall suicide deaths [7], its exact impact on the temporal patterns of charcoal burning suicides is unclear. It is reasonable to assume that the employed victims were particularly vulnerable to the fear of job loss, which has been found to significantly predict depressive symptoms and suicide mortality [18,19]. As the weekend represents a potential break from the stresses experienced during the work week, it is possible that suicide rates are lower during the weekend than during the work week. Employed victims may also experience a greater fear of job loss during seasonal layoff periods and thus may be more inclined to commit suicide.

The unemployed may also have a higher suicide risk at specific times as a result of the stress of job searching. Some researchers have noted that during weekends, the unemployed tended to spend more time on socializing activities and significantly less time on job searching [20]. Consequently, unemployed people may be more likely to commit suicide during the week, as they experience higher levels of stress in relation to job searching during this period of time [20]. The unemployed may also be more anxious about finding a job during recruitment seasons. Nonetheless, community-based research in this area is lacking. The influence of economic activity status on the daily and monthly patterns of charcoal burning suicides is yet to be examined.

In addition to economic activity status, sex difference may also influence the temporal pattern of charcoal burning suicides. In previous studies, a bimodal distribution of monthly suicide deaths was observed in women but not in men [21,22]. Sex also interacted with the suicide method on the temporal patterns of suicide [17,23]. As the victims of charcoal burning suicide were predominately economically active men [1,2], an interaction between sex and economic activity status on the temporal pattern of charcoal burning suicides is possible. However, this is yet to be investigated.

The present study aims to fill these gaps in the existing research by examining the temporal asymmetries in the monthly and daily distributions of charcoal burning suicides in Hong Kong. In particular, the study will examine the temporal variations of charcoal burning suicides in relation to the economic activity status and sex of victims of usual working age (20–59).

## Methods

The present study covers the period, from 2001 to 2008. Suicide data were obtained from the registered death files of the Census and Statistics Department of Hong Kong. Following the 10th Revision of the International Classification of Diseases and Related Health Problems (ICD–10), reportable deaths with an external cause code ranging from X60 to X84 are classified as suicide [24]. A total of 8165 suicide cases were reported during the overall study period, with 5500 cases (67.4%) involving victims of usual working age (20–59). Of these 5500 cases, 30.0% (1649 cases) were charcoal burning suicides (X67). All of the recorded suicides included information on the age, sex, and economic activity status (working versus non-working) of the victim, and the date of death and method used.

To analyze the monthly and daily patterns, the data were divided into 12 months of a year and 7 days of a week, respectively. To examine the amplitude of temporal variation in suicides and its statistical significance, multinomial Poisson regression analyses were conducted by fitting the number of suicides for each month/day of the week with the corresponding time variable [10,25]. Given that the size of the usual working age population in Hong Kong increased by 10% during the study period, from 3.08 million in 2001 to 3.33 million in 2008, the Poisson regression is a more appropriate method than the Chi-square test, which assumes that there has been no significant change in the size of the population. The Poisson regression can also adjust for changes in regard to sex and economic activity status within the population over the study period.

To adjust for differences in age and sex in the suicide rate and population structure, we included the age, sex, and economic activity status of the suicide victims, the year of incidence and the offset term of population size in the regression equation as the confounding variables. Mathematically, the regression equation is written as follows:

(1) $$\text{log}\left(\text{suicides}\right)=\alpha +\sum_{i=1}\beta_{i}\left({\text{X}}_{i}\right)+\delta_{1}\left(\text{Sex}\right)+\delta_{2}\left(\text{age}\right)+\delta_{3}\left(\text{Economic activity}\right)+\delta_{4}\left(\text{year}\right)+\text{ log}\left(\text{Population}\right)\;$$

The temporal variation was modeled by comparing the actual frequencies of suicides with the expected values based on the assumption that the suicides are equally distributed. The exponential of the regression coefficient (β) represents the incidence risk ratio (IRR), which describes the multiplicative effect of the corresponding independent variable on the risk [26-28]. A significant IRR indicates that there is a significant difference in the adjusted suicide risk between the corresponding month (or day of the week) and the reference period of time. In the present study, the suicide risks for December and Saturday (which previous studies reported as being the periods of lowest risk [13-15]) were used as references to examine the respective monthly and daily patterns of suicide.

In the present study, the seasons were defined as follows: spring (from March to May), summer (from June to August), autumn (from September to November) and winter (from December to February) [13]. In all statistical analyses, a p–value smaller than 5% was considered to be statistically significant. All of the statistical work was performed using the SAS statistical software package for Windows, version 9.1.

## Results

### Social demographic profile of victims

Of the 1649 charcoal burning suicide victims, 68.2% (n = 1124) were men and 31.8% (n = 525) were women. The proportions of those who were working and non-working were 42.5% (n = 701) and 53.2% (n = 878), respectively. The economic activity status of the remaining 4.3% (n = 70) of victims was unknown. Only those who were recorded as either working or non-working were included in the analyses. Thus, a total of 498 working men, 203 working women, 571 non-working men, and 307 non-working women were included in the analysis of the influence of economic activity status and sex on the temporal variation in charcoal burning suicides.

### Monthly pattern of suicides

Overall, moderate temporal variations were observed in the monthly patterns of charcoal burning suicides (Table1). A peak was observed in April (spring) (IRR = 1.389, p = 0.0049), but only among those who were working (IRR = 1.559, p = 0.0106). Significant sex differences were also observed. Whereas men were less likely to commit suicide in summer (June: IRR = 0.70, p = 0.0228; July: IRR = 0.73, p = 0.047), women were more likely to commit suicide in spring (April: IRR = 1.72, p = 0.0186 and May: IRR = 1.60, p = 0.0434), late autumn (November: IRR = 1.65, p = 0.0307), and winter (January: IRR = 1.97, p = 0.0026; February: IRR = 1.62, p = 0.0406).

**Table 1:** Incidence risk ratios of suicide for deceased aged 25–59 by month, Hong Kong, 2001-2008

	**Working (n = 701)**			**Non-working (n = 878)**			**Overall (n = 1649)**^**a**^		
	**Men (n = 498)**	**Women (n = 203)**	**All**	**Men (n = 571)**	**Women (n = 307)**	**All**	**Men (n = 1124)**^**b**^	**Women (n = 525)**^**c**^	**All**
January	1.14	2.55 *	1.418	0.91	1.63	1.096	1.01	1.97 *	1.234
February	0.78	1.91	1.007	0.92	1.46	1.062	0.86	1.62 *	1.038
March	1.23	1.73	1.327	1.07	1.05	1.068	1.14	1.3	1.18
April	1.43	2.07 *	1.559 *	1.17	1.52	1.26	1.29	1.72 *	1.389 *
May	0.98	2.18 *	1.218	1.04	1.26	1.096	1.01	1.60 *	1.148
June	0.85	1.13	0.902	0.57 *	1.31	0.764	0.70 *	1.24	0.823
July	0.68	1.27	0.8	0.78	1.63	1	0.73 *	1.5	0.914
August	0.89	1.36	0.982	0.83	1	0.877	0.86	1.13	0.922
September	0.7	1.32	0.827	0.77	1.47	0.948	0.74	1.41	0.896
October	0.89	1	0.909	0.69	1.21	0.822	0.78	1.13	0.859
November	0.87	1.32	0.958	0.96	1.85 *	1.189	0.92	1.65 *	1.09
December ^#^	1	1	1	1	1	1	1	1	1

*significant at 5% level.

^#^ reference group.

^a^ 70 deceased had unknown economic activity status.

^b^ 55 male deceased had unknown economic activity status.

^c^ 15 female deceased had unknown economic activity status.

The interaction of sex and economic activity status was also observed in the monthly pattern of charcoal burning suicides. Although no monthly variation was observed in the suicide rate of working men, working women were more inclined to commit suicide in spring (April: IRR = 2.07, p = 0.0493 and May: IRR = 2.18, p = 0.0321) and winter (January: IRR = 2.55, p = 0.0086). As for the non-working victims, men were less likely to commit suicide in summer (June: IRR = 0.57, p = 0.0148), whereas women were more likely to commit suicide in late autumn (November: IRR = 1.85, p = 0.0319).

### Daily pattern of suicides

Significant temporal variations were observed in the daily patterns of charcoal burning suicides (Table2). In general, there were excess risks of suicide on Monday (IRR = 1.633, p < .0001), Tuesday (IRR = 1.367, p = 0.0028), and Thursday (IRR = 1.266, p = 0.0268). However, the peaks on Monday (IRR = 1.806, p = 0.0001) and Tuesday (IRR = 1.507, p = 0.0092) were only noted in those who were working, whereas there was only a single peak on Monday (IRR = 1.505, p = 0.0025) for those who were non-working. Slight variations were observed in regard to the sex of the victims. Both sexes had peaks on Monday (Men: IRR = 1.64, p = <.0001; Women: IRR = 1.61, p = 0.0113) and Tuesday (Men: IRR = 1.31, p = 0.0302; Women: IRR = 1.50, p = 0.0332), though women had an additional peak on Thursday (IRR = 1.57, p = 0.0176).

**Table 2:** Incidence risk ratios of suicide for deceased aged 25–59 by day of the week, Hong Kong, 2001-2008

	**Working (n = 701)**			**Non-working (n = 878)**			**Overall (n = 1649)**^**a**^		
	**Men (n = 498)**	**Women (n = 203)**	**All**	**Men (n = 571)**	**Women (n = 307)**	**All**	**Men (n = 1124)**^**b**^	**Women (n = 525)**^**c**^	**All**
Sunday	1.11	1.45	1.209	1.00	1.38	1.11	1.04	1.41	1.152
Monday	1.91 *	1.55	1.806 *	1.45 *	1.65 *	1.505 *	1.64 *	1.61 *	1.633 *
Tuesday	1.68 *	1.10	1.507 *	1.05	1.81 *	1.264	1.31 *	1.50 *	1.367 *
Wednesday	1.17	1.50	1.269	1.11	1.31	1.165	1.13	1.39	1.209
Thursday	1.21	1.35	1.254	1.09	1.73 *	1.275	1.14	1.57 *	1.266 *
Friday	1.30	1.05	1.224	1.00	1.54	1.154	1.13	1.33	1.184
Saturday ^#^	1	1	1	1	1	1	1	1	1

* significant at 5% level.

^#^ reference group.

^a^ 70 deceased had unknown economic activity status.

^b^ 55 male deceased had unknown economic activity status.

^c^ 15 female deceased had unknown economic activity status.

An interaction between sex and employment status on the daily pattern of charcoal burning suicides was noted. For those who were working, excess risks of suicide were observed only in men (Monday: IRR = 1.91, p = 0.0003; Tuesday: IRR = 1.68, p = 0.0048). Among those who were non-working, men had a single peak on Monday (IRR = 1.45, p = 0.0222), whereas women had multiple peaks, on Monday (IRR = 1.65, p = 0.0429), Tuesday (IRR = 1.81, p = 0.0154), and Thursday (IRR = 1.73, p = 0.026).

## Discussion

To our knowledge, the present study is the first to investigate the temporal patterns of charcoal burning suicide. As charcoal burning is a non-violent suicide method that requires planning to avoid rescue intervention [1-3], the temporal variations in suicide observed in this study contradict the findings of many published studies that suicide seasonality only exists in relation to violent methods [8,16,17]. This suggests that the specific suicide method is an important factor of suicide seasonality, and that the simple differentiation between violent and non-violent methods is not applicable to the interpretation of suicide seasonality in Hong Kong [13]. In fact, a range of published studies have demonstrated that temporal variations in suicides are method-specific [13,29,30]. Future research on temporal patterns of suicide should be more method-specific.

Previous studies have attributed the monthly patterns observed in suicide rates to the influence of seasonality-linked biological, psychosocial and meteorological correlates [12,16,17,31]. As the April peak in the seasonality of suicide has also been observed in other suicide methods, charcoal burning suicides may share some of the common causes of suicides by other methods. In addition to the presence of spring peak in April, it is observed that attempters were more likely to commit suicide by burning charcoal in the colder winter months. From a practical point of view, the sub-tropical climate of Hong Kong would implicitly discourage suicide attempters from burning charcoal in summer to take their own lives. By contrast, the cooler temperatures in winter would make charcoal burning a more acceptable option for suicide. However, the extent to which these explanations are able to account for the monthly pattern of charcoal burning suicides remains unclear. Some studies suggest that the correlation between biological factors and seasonal variation is only observed in violent suicides [32,33]. Furthermore, the findings on the effects of meteorological variables on suicide seasonality are rather inconsistent and have not been replicated in subsequent studies [10,34]. It is worth noting that only employed women tended to commit suicide by burning charcoal in spring (i.e., April). Hence, the seasonal occurrence of particular mental disorders involving suicide ideation fails to explain the temporal variations in charcoal burning suicides. In fact, this is in line with the observation that charcoal burning suicide victims were less likely to be associated with psychiatric illness or substance abuse, compared with those who killed themselves using other methods [1-5]. We expect that more detailed data on the events prior to the death of charcoal burning suicide completers, and on what the suicide attempters were thinking, will help us to explore this knowledge gap in future studies.

The results confirm our hypothesis concerning the daily pattern of charcoal burning suicides. The risk of charcoal burning suicide was found to be higher on weekdays (i.e., Monday, Tuesday, and Thursday) than on weekends. This is consistent with most of the other published findings, which report a peak at the beginning of the week [14,15,35,36]. As suggested by Erazo et al. [36], a significantly higher rate of suicide on Monday seems to be best explained in terms of socio–psychological variables. Relatively speaking, a sense of personal failure and isolation is more likely to be triggered in a depressive person at the beginning of the working week, when their surroundings reflect their duties [36]. A recent study on the relationship between the weekend, work, and well-being also indicated that both men and women tended to experience an enhanced sense of well-being during the weekend compared with working days [37]. Yet, the second peak on Thursday observed in the present study is rather novel in the literature. Further investigation of the relationship between weekdays and psychological stress is warranted.

In line with our expectations, there were pronounced differences in the temporal patterns of charcoal burning suicides in relation to sex. The results identified marked peaks in the rates of charcoal burning suicide in spring and winter (January, February, April, May and November) for working-age women, a less obvious spring peak with a summer nadir was observed for working-age men. This seasonal difference in the magnitude of variation] may be further explained by gender differences in relation to thermal comfort. In an earlier physiological study, Lan et al. [38] found that Chinese women were more sensitive to temperature differences than men and that women preferred a relatively warmer environment because they had a higher comfortable operative temperature. It can thus be expected that women are more inclined to burn charcoal in “cold months” and men are less likely to do so in “hot months”. As our knowledge of the determinants of charcoal burning suicide remains scant, further research is needed to investigate the differential effects of charcoal burning on vulnerable men and women in terms of monthly patterns.

It is worth noting that the influence of employment status on monthly and daily patterns of charcoal burning suicides differed considerably between men and women. According to our data, the monthly variations in suicide risk were more pronounced for non-working men and working women, whereas the daily variations were more apparent for working men and non-working women. It appears that economic activity status either strengthens or weakens the magnitude of the temporal variations in charcoal burning suicide, rather than serving as a determinant that alters the temporal distribution. This may indicate that active employment only serves as a stabilizing factor that protects individuals from various life-threatening forms of behavior (e.g. alcohol consumption or misuse of illicit drugs) [39] and thereby implicitly influences the timing of suicide among specific groups of individuals, but not the whole population. Accordingly, the nature and magnitude of these effects should be recognized in future studies to develop a more effective approach to suicide prevention in Hong Kong.

## Conclusions

Our findings contribute to the existing suicide research by extending our scientific knowledge of the temporal patterns of suicidal behavior. In practice, the results of this study may increase the awareness of clinicians, volunteers, and other stakeholders of the most probable timings of suicide, when at-risk individuals may be more vulnerable to suicidality [36,40,41]. A greater understanding of the temporal patterns of suicidal behavior may contribute to establishing more effective suicide prevention strategies [36]. In particular, the different patterns of suicide risk observed in each subgroup suggest that intervention practices for charcoal burning suicides should be more group-specific.

Several caveats on our findings need to be mentioned. First, the present study failed to separate the unemployed victims from the economically inactive victims due to the limited information available from the dataset. The employed and unemployed victims were expected to have similar variations in suicide risk, whereas the unemployed and economically inactive victims were likely to commit suicide at different times [42]. Future research should independently examine the temporal patterns of all three groups to gain a more comprehensive picture of the influence of economic activity status on suicide risk among charcoal burning victims. Second, we only investigated the temporal variation in charcoal burning suicides among the usual working-age population (20–59). Our study did not consider the situation of older adults. Nonetheless, older adults comprised a relatively insignificant proportion of the total number of charcoal burning suicides (n = 90) over the study period. Third, the present study did not include individual information on alcohol consumption, history of substance abuse or mental disorders, which are all known to heighten the risk of suicide [10,30,43-45]. From an epidemiological perspective, seasonal changes in the course of depressive disorder, its treatment indices, and suicide, all appear to coincide and indicate that depressive episodes are an important determinant of the temporal variation in suicide [8-10,12]. To provide a more complete picture of temporal variation in suicide, researchers should also examine the mental health background of suicide victims. Future analyses should include these variables to develop a more comprehensive surveillance and monitoring system for detecting suicidal behavior.

## Competing interests

All authors declare that they have no competing interests.

## Authors’ contributions

CKL (the leading author) designed the study, formulated the research questions, undertook the statistical analyses and wrote the first draft of the manuscript. CMCL managed the literature search and review and provided comments during the final stage of manuscript preparation. Both authors were responsible for drafting and revising this manuscript and have read and approved the final version.

## Pre-publication history

The pre-publication history for this paper can be accessed here:

http://www.biomedcentral.com/1471-2458/12/505/prepub
